# Rennrodel-Hochrasanztrauma mit Komplextrauma des Fußes und akutem Kompartmentsyndrom eines 8-jährigen Kindes: Ein 18-Jahres-Spätergebnis

**DOI:** 10.1007/s00113-026-01718-7

**Published:** 2026-06-02

**Authors:** Hans Zwipp, Achim Biewener, Guido Fitze, Stefan Rammelt

**Affiliations:** 1 UniversitätsCentrum für Orthopädie, Unfall- und Plastische Chirurgie am Universitätsklinikum Carl Gustav Carus, Technische Universität Dresden, Fetscherstraße 74, 01307 Dresden, Deutschland; 2 Klinik und Poliklinik für Kinderchirurgie im Universitäts Kinder-Frauenzentrum am Universitätsklinikum Carl Gustav Carus, Technische Universität Dresden, Fetscherstr. 74, 01307 Dresden, Deutschland

**Keywords:** Multiple Frakturen am Fuß, Intraartikuläre Kalkaneustrümmerfraktur beim Kind, Akutes Fuß-Kompartmentsyndrom beim Kind, Dorsomediane Fasziotomie, Tibiometatarsale Transfixation, Multiple fractures at the foot, Intra-articular comminuted calcaneal fracture in children, Acute foot compartment syndrome in children, Dorsomedian (central) fasciotomy at the foot, Tibiometatarsal transfixation

## Abstract

**Hintergrund:**

Das Komplextrauma des Fußes mit akutem Kompartmentsyndrom (KS) beim Kind ist eine absolute Rarität, für welche in der Literatur kaum Therapieempfehlungen vorliegen.

**Ziel der Arbeit:**

Aufzeigen einer schrittweisen Versorgung, die trotz der Schwere des Knochen‑, Gelenk- und Weichteiltraumas ein sehr gutes Spätergebnis ergab.

**Material und Methode:**

Beim Rennrodel-Training prallte ein 8‑jähriges Kind mit ca. 90 km/h mit dem linken Fuß gegen die Bande, was zu einem Komplextrauma des Fußes (6 Punkte nach Zwipp) mit serieller Fraktur des Talus, Kalkaneus (5 Fragment/3 Gelenk mit 50°-Varus-Fehlstellung), Kuboid und Metatarsale V mit additivem akuten Fuß-KS führte. Eine 3‑Schritte-Versorgung mit akuter dorsomedianer Kompartmentspaltung („Hannover approach“) plus tibiometatatarsaler Transfixation (Tag 1), intermediärer Hautadaptation (Tag 5) und definitiver offener, anatomischer Gelenkrekonstruktion des Kalkaneus (Tag 8) ermöglichte einen komplikationslosen Heilverlauf. Sechs Wochen nach der 3. Operation folgten die Entfernung des Unterschenkelgipsverbandes und der K‑Drähte. Kein Schulsport für 10, intermittierende Physiotherapie für 12 Monate.

**Ergebnis:**

Der Patient ist 18 Jahre nach Komplextrauma des linken Fußes als Lackier-Meister im 3‑Schicht-System uneingeschränkt arbeitsfähig. Fußball- und Kraftsport sind schmerzfrei möglich bei voller OSG-, Fuß- und Zehenfunktion. Der American Orthopaedic Foot & Ankle Society (AOFAS-) Score beträgt 100, der Foot Function Index 2,36 und der EuroQol-5L5D-Score 100 Punkte. Ein CT im Stand zeigt nach 18 Jahren gut konservativ-verheilte Frakturen von Talus, Kuboid und Metatarsale V sowie einen in Form und Achse anatomisch wiederhergestellten operativ versorgten Kalkaneus mit arthrosefreien und kongruenten posterioren, medialen und kuboidalen Gelenkfacetten.

**Diskussion:**

Die dringliche Entlastung des akuten Fuß-KS möglichst innert 6 h nach Trauma ist auch beim Kind unerlässlich. Ein abgestuftes Vorgehen beim komplexen Fußtrauma ist empfehlenswert.

## Abstract (en)

**Background:**

Complex trauma of the foot with acute compartment syndrome (CS) in children is an absolute rarity for which there are barely any treatment recommendations in the literature.

**Objective:**

Presentation of a staged operative management that yielded an excellent long-term result despite severe bone, joint and soft tissue injuries.

**Material and method:**

During luge training an 8‑year-old child collided with the barrier at approximately 90 km/h with the left foot, resulting in complex trauma to the foot (6 points according to Zwipp) with serial fractures of the talus, calcaneus (5 fragments/3 joints) with 50° varus deformity, cuboid and fifth metatarsal with acute foot CS. A 3‑stage treatment consisting of acute dorsomedial compartment release (Hanover approach) plus tibiometatarsal external fixation (day 1), intermediate skin adaptation (day 5) and definitive open anatomical joint reconstruction of the calcaneus (day 8) enabled an uneventful healing process. The below knee cast and K‑wires were removed 6 weeks after the third surgery. The child was exempted from physical education for 10 months and received intermittent physiotherapy for 12 months.

**Results:**

The patient, 18 years after the complex trauma of the left foot, is working full time as an industrial master painter in a 3-shift system. He can play soccer and do weight training without pain with full ankle, foot and toe function. The American Orthopaedic Foot & Ankle Society (AOFAS) ankle hindfoot score at 18 years follow-up is 100, the foot function index is 2.36, and the EuroQoL-5L5D score is 100 points. Weight-bearing computed tomography (CT) shows conservatively treated well-healed fractures of the talus, cuboid and fifth metatarsal. The anatomically realigned calcaneus is free of arthrosis with congruent posterior, medial and cuboid joint facets.

**Discussion:**

Urgent relief of acute foot CS within 6 h of trauma is also essential in children. A staged surgical approach is recommended for complex foot trauma with severe soft tissue injury.

## Einleitung

Fersenbeinbrüche sind unter allen Frakturen beim Kind mit 0,005–0,15 % sehr selten, ereignen sich am ehesten im Alter von 8 bis 12 Jahren, meist beim Sturz aus der Höhe oder als Verkehrsunfall [9, 20, 23]. Bei alleiniger Betrachtung der Frakturen an oberem Sprunggelenk (OSG) und Fuß, ohne die sehr häufigen Zehenbrüche, sind nach eigener Analyse im Kindesalter das OSG in 53,9 %, die Metatarsalia in 28,1 %, der Talus in 6,3 % und das Fersenbein in 4,7 % am häufigsten betroffen [30]. Ein akutes Kompartmentsyndrom (KS) des Fußes wird bei Kindern (2 bis 14 Jahre) als äußerst seltene Diagnose mit 0,00002 % [6] angegeben. Während die Kombination von Kalkaneusfraktur und akutem Fuß-KS beim Erwachsenen in 1–10 % der Fälle beobachtet wird [14, 17, 26, 27], ist diese beim Kind eine absolute Rarität. Dies gab Anlass zur vorliegenden Fallvorstellung, da ein zu spät oder unerkanntes akutes Fuß-KS beim Kind wie beim Erwachsenen zu Hammer‑, Krallenzehen, sogar zu schwerer posttraumatischer Hallux-valgus-Deformität führen kann [13, 22, 32].

## Unfallhergang

**Abb. 1:**
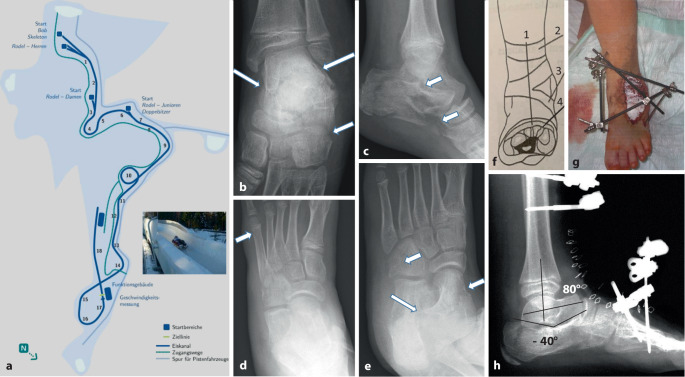
**a–h***Unfalltag:* Rennschlitten- und Bobbahn Altenberg (**a**). Start kurz vor Kreisel (*10*), Unfall nach Kurve *15*. **b–e** Unfallröntgenbilder: *Pfeile* zeigen auf die Talus-Kalkaneus-Kuboid-Metatarsale-V-Fraktur. **f–h***Notfalloperation:* akute Kompartmentspaltung (**f**) mit Spaltung von Haut (*1*), proximalem Extensoren-Retinaculum (*2*), distalem Extensoren-Retinaculum (*3*), Fascia dorsalis pedis (*4*); tibiometatarsaler Minifixateur (**g**) mit Kunsthautdeckung (Foto erstellt von der Mutter beim Verbandwechsel am 2. Tag). **h** Seitliches Röntgenbild: negativer Böhler-Winkel (−40° statt zwischen 25 und 45°) und ein tibiotalarer Winkel von 80° (statt 110°). Liegende Klammern nach Kunsthautdeckung

Ein 8‑jähriges Mädchen verunfallte beim Training auf der Rennschlitten- und Bobbahn Altenberg am Spätnachmittag im Februar 2008 (Abb. 1a). Nach dem Start etwas oberhalb des Kreisels geriet der Schlitten nach der drittletzten Linkskurve durch eine Unregelmäßigkeit in der Eisbahn ins Rutschen. Die junge Fahrerin steuerte gegen, verlor die Kontrolle und prallte bei ca. 90 km/h ungebremst mit dem linken Fuß gegen die steile Bande und überschlug sich danach mehrfach.

## Erstversorgung, Diagnostik, Verlegung und Notoperation

**Abb. 2:**
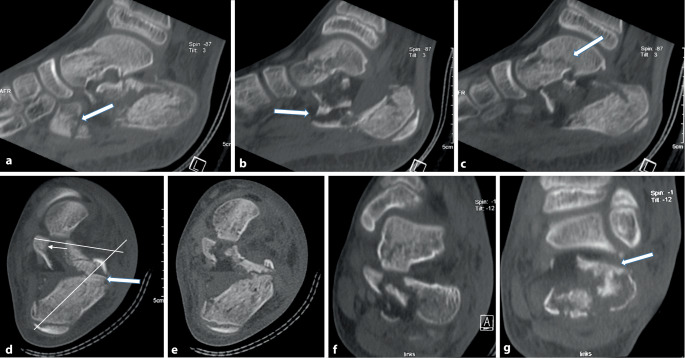
*CT-Analyse:* komplexes Fußtrauma mit mäßig dislozierter Kuboidfraktur (**a**, *weißer Pfeil*), mehrfragmentärer Fraktur des Processus anterior calcanei mit Dislokation der Gelenkfläche zum Kuboid (**b**, *weißer Pfeil*), minimal dislozierter Talushals/Taluscorpus-Fraktur (**c**, *weißer Pfeil*), um 50° varisch disloziertem Tuber calcanei sowie Fraktur der medialen (**d–f**) und posterioren (**g**) Gelenkfacette des Subtalargelenks

Bei klinisch sicherer Verletzung des linken distalen Unterschenkels und Fußes erfolgte die Erstversorgung des bewusstseinsklaren Kindes mittels flexibler Schiene und Eispackung. Im nahegelegenen Krankenhaus erfolgte eine Röntgendiagnostik (Abb. 1b–e) sowie, wegen des Komplextraumas, die Verlegung ins Universitätsklinikum. Hier wurde bei extremer, schmerzhafter Schwellung des linken Fußes mit Taubheit zwischen der 1. und 2. Zehe die klinische Diagnose eines akuten Fuß-Kompartmentsyndroms (KS) bei verletzungsführender 3.° geschlossener Fersenbeintrümmerfraktur gestellt. Auf eine Druckmessung wurde bei eindeutigen klinischen Zeichen verzichtet, vielmehr ein Notfall-CT des linken Fußes (Abb. 2a–g) sowie eine Notoperation indiziert, die noch vor Mitternacht in Allgemeinnarkose erfolgte. Dabei wurde unter Verzicht auf eine Blutsperre der „Hannover approach“ [31] mit dorsomedianer Längsinzision der Haut von ca. 13 cm Länge (Abb. 1f, g), Längsspaltung der Fascia dorsalis pedis sowie des proximalen und distalen Extensoren-Retinaculums zur Entlastung des isolierten Fuß-KS gewählt. Nach subtiler Blutstillung wurde der entstandene spindelförmige, ca. 13 × 6 cm große Hautdefekt (Abb. 1g) mit Kunsthaut aus Teflon/Polyurethan (EpiGARD®, Fa. Biovision, Ilmenau) gedeckt. Danach erfolgte die Ruhigstellung der Weichteile bei Komplextrauma des Fußes im tibiometatarsalen Minifixateur. Dazu wurden zwei 3,0-mm Schanz-Schrauben in die ventrale, distale Tibia und je eine 2,5-mm Schanz-Schraube in das erste und in das basisnahe 5. Metatarsale (lateral der undislozierten Spiralfraktur) gesetzt, die Stabilität der Montage mit Karbon-Längsträgern zur Transfixation des Fußes in Rechtwinkelstellung erzielt (Abb. 1d, g, h).

## Operativer Schritt 2

**Abb. 3:**
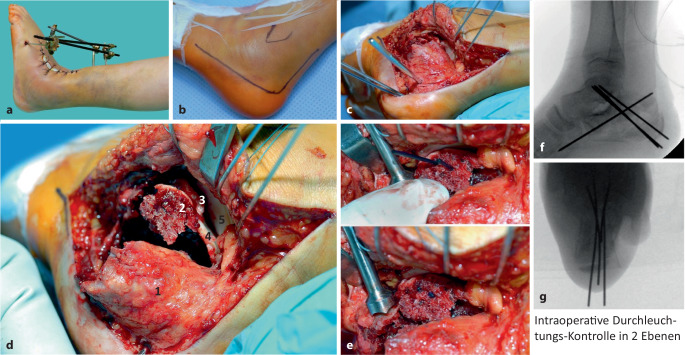
*Dritter operativer Schritt* (8. Tag) **a** Fixateur-Entfernung, Hautnaht **b** sterile Kompressen- u. Folienabdeckung mit Einzeichnen des lateralen Zugangs. **c** Laterales Setzen einer Schanz-Schraube ins Tuber calcanei zur Varuskorrektur. **d** Nach Lösen der seitlichen Wand (*1*), des impaktierten posterioren Facetten(pf)-Fragmentes (*2*) werden ein drittes, 180° luxiertes pf-Fragment (*3*) erkennbar sowie das größte, abgesunkene und unterminierte pf-Fragment (*4*) erkennbar, das kongruent zum talaren Knorpel (*5*) angehoben werden muss, mit Fixation der 3 pf-Fragmente mit resorbierbaren Stiften (*blau*), schrittweise gekürzt (**e**) mit abschließender Reposition der lateralen Wand, Lösen und Achsenkorrektur des Tuber calcanei und Fixation mit 4 perkutanen K‑Drähten (**f,g**)

Am 5. Tag nach dem Unfall erfolgte der *2. operative Schritt* mit Kunsthautwechsel und Adaptation der Wundränder am Fußrücken auf jetzt 12 × 2 cm EpiGARD®-Deckung (Abb. 3a) in kurzer Narkose.

## Indikationsfindung zum 3. operativen Schritt

Entsprechend der CT-Analyse (Abb. 2a–g) entsprach die Kalkaneusfraktur nach CT-Klassifikationen bei Erwachsenen einer *5-Fragment-3-Gelenk-Fraktur* [22] oder nach Sanders einem Typ IV [21]. Aufgrund des drittgradigen Weichteilschadens (g3) nach Oestern und Tscherne [18] waren zur Definition eines *Komplextraumas des Fußes* (≥ 5 Punkte) [31] bereits 3 Score-Punkte gegeben, aufgrund der seriellen Fraktur auf 3 Ebenen (Talus-Kalkaneus-Chopart) 3 weitere Score-Punkte, sodass mit 6 Punkten ein Komplextrauma vorlag. Nach der prognostischen Fersenbeinbeinfrakturskala [29, 31] kamen zu den 5 Hauptfragmenten und 3 betroffenen Gelenken für den drittgradigen Weichteilschaden 3 weitere Punkte und ein 12. Punkt für die Talus- und Kuboidfraktur hinzu, sodass mit 12 von 12 Punkten eine schlechte Prognose gegeben war. Deshalb wurde der Mutter im Aufklärungsgespräch erklärt, dass bei sehr niedrigem Böhler-Winkel, erheblicher Fragmentierung und Dislokation des Subtalargelenkes sowie 50°-varischer Fehlstellung des Tuber calcanei ein operatives Vorgehen mit ausgedehnt lateralem Zugang einem Versuch zur Schadensbegrenzung gleichkäme. Auch sollten wegen des schweren Weichteilschadens bei minimaler Dislokation der Talushals‑/Taluscorpus-Fraktur sowie der mäßig im Gelenk dislozierten Kuboidfraktur auf deren Fixierung verzichtet und nur das schwer zerstörte Fersenbein adressiert und möglichst mit einem Minimum an Fremdmaterial im Sinne einer Kirschner-Draht-Osteosynthese stabilisiert werden.

## Operativer Schritt 3

Am 8. Tag nach dem Unfall konnte nach Abschwellung der Weichteile zur definitiven Fersenbeinversorgung der *3. operative Schritt* erfolgen. Dabei wurden in Intubationsnarkose zunächst der Fixateur externe entfernt und unter sterilen Kautelen und Rechtsseitenlage die restliche Kunsthaut abgelöst, die Fußrückenwunde gespült, die Wundränder angefrischt und vernäht und diese sowie die Pin-Eintrittsstellen mit Kompressen und Folie abgedeckt. Danach erfolgte der ausgedehnt laterale Zugang zum Fersenbein (Abb. 3b). Nach senkrechter Inzision von Haut und subkutaner Fettschicht wurden diese gewebeschonend mit Fäden angeschlungen und schrittweise retrahiert, das Subtalargelenk durch Setzen von drei 1,4-mm K‑Drähten in den Taluskörper dicht oberhalb der Facies articularis calcanea posterior exponiert. Zur Varuskorrektur wurde eine Schanz-Schraube dorsal der weit nach lateral ausgebuckelten Wand ins Tuber calcanei gesetzt (Abb. 3c). Das nach dorsal um 1 cm luxierte Processus-anterior-Fragment wurde von den Weichteilen gelöst und nach plantar reponiert. Die Darstellung der dreifach gebrochenen posterioren Facette (pf) gelang nach Lösen der seitlichen Wand und des in ihr impaktierten pf-Fragmentes, woraufhin ein um 180° luxiertes pf-Fragment erkennbar wurde sowie das größte, abgesunkene, schalenförmige posterolaterale pf-Fragment kongruent zum talaren Knorpel angehoben wurde (Abb. 3d). Die in sich gespaltene mediale Facette und die drei Fragmente der posterioren Facette wurden von medial nach lateral kongruent zum Talus hin und zueinander reponiert, mit 1,4-mm K-Drähten temporär gehalten und schrittweise durch 3 resorbierbare Polydioxanon (PDS)-Stifte (Ethipin®, Fa. Wörner Medical, Reutlingen) ersetzt (Abb. 3e). Danach wurde der Stauchungsdefekt mit lokaler Spongiosa unterfüttert, die massiv in das Tuber impaktiert war. Mit abschließender Reposition der lateralen Wand, sukzessivem Lösen und Achsenkorrektur des Tuber calcanei konnte dieses schließlich mit 3 perkutan eingebrachten 1,6-mm K-Drähten zum Talus (Abb. 3f, g) fixiert werden, wobei ein 4. K-Draht bereits zuvor zur Fixation des reponierten Processus-anterior-Fragmentes platziert worden war. Das Schwierigste der 2 h und 5 min dauernden Operation, mit nur initial kurzfristig angelegter Blutsperre, war das Lösen des extrem varisch abgewinkelten, tief impaktierten Tuber calcanei mit einer wegen der noch offen Fersenapophyse von lateral eingebrachter Schanz-Schraube (Abb. 3c). So mussten zur Fixation die perkutan eingebrachten und später unter Hautniveau gekürzten 1,6-mm-Kirschner-Stahl-Drähte zur Gewährleistung einer anatomischen Achsenkorrekter 2‑mal nachkorrigiert werden (Abb. 3f, g). Nach Spülung, Einlegen eines Kollagenschwammes und einer Redon-Drainage erfolgten Hautnaht, steriler Verband, ein Unterschenkelspaltgips und die Lagerung in einer Volkmann-Schaumstoff-Schiene.

## Nachbehandlung

**Abb. 4:**
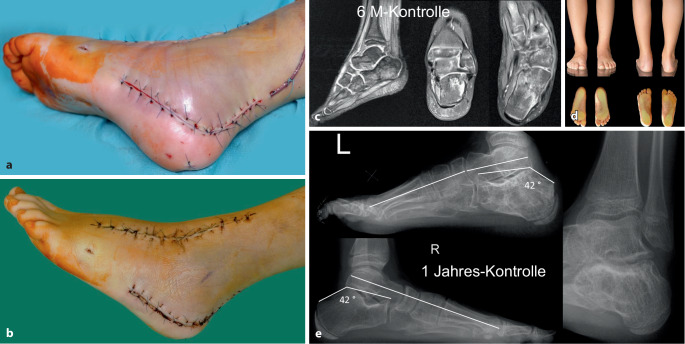
Weichteil‑, MRT- und Röntgenkontrollen: **a** Weichteile unmittelbar nach Fersenbeinrekonstruktion. **b** Weichteile 5 Tage später. **c** MRT 6 Monate postoperativ mit guter Kongruenz des Subtalar- und Kalkaneokuboidalgelenkes. **d** Spiegeltischaufnahme nach 6 Monaten ohne Zehendeformität links nach abgelaufenem KS. **e** 1-Jahres-Röntgen stehend: Böhler-Winkel beidseits 42°, Prominenz des Taluskopfes links, 2–3 mm Verkürzung des Os cuboideum mit plantarer Ausziehung bei harmonischer Cyma-Linie (Chopart-Gelenk). Physiologische Talometatarsale-Achse links. In der 20°-Brodén-Projektion physiologische Rückfußachse und kongruentes Subtalargelenk

Bei primärer Wundheilung (Abb. 4a, b) wurden nach 10 Tagen die Fäden entfernt, ein geschlossener Gehgipsverband angelegt und das Kind nach Gehübungen mit 2 Unterarmgehstützen in die Häuslichkeit entlassen. 6 Wochen später wurden der Gipsverbandes und die 4 Kirschner-Drähte in Kurznarkose entfernt. Es folgten ambulante Gehschulung und Krankengymnastik.

## 18-Jahres-Verlauf

*6 Monate* nach letzter Operation kommt die Patientin in normalem Schuhwerk ohne Einlagen gut zurecht, fühlt sich noch gangunsicher, hat keine relevanten Schmerzen beim Gehen, ist noch vom Schulsport befreit und erhält noch eine spezielle Physiotherapie. Sprunggelenk‑, Fuß- und Zehenfunktion sind gut. Bei dem jetzt 9‑jährigen Mädchen zeigen MRT- und Spiegeltischaufnahmen (Abb. 4c, d) gute Knorpelverhältnisse im Subtalar- und Kalkaneokuboidalgelenk sowie keine Folgen im Zehenbereich nach Fuß-KS links.

*12 Monate* nach dem Unfall nimmt das Kind seit 2 Monaten wieder am Schulsport teil und klagt lediglich über Narbenempfindlichkeit. Die Röntgenbelastungsaufnahmen und die 20°-Brodén-Projektion (Abb. 4e) zeigen einen seitengleichen Böhler-Winkel von 42°, ein gutes Subtalargelenk mit physiologischem Rückfußvalgus von 7° und eine nahezu seitengleiche talometatarsale Fußachse.

*18 Jahre* nach dem Unfall gibt der inzwischen 26-jährige Patient an, 4 Jahre zuvor eine Geschlechtsumwandlung absolviert zu haben, welche in keinem Zusammenhang mit dem durchgemachten Unfall gestanden hätte. Er berichtet, dass er einen empathischen und geduldigen Physiotherapeuten hatte, der ihm die Angst vor dem normalen Gehen nahm. Schuheinlagen wurden nicht benötigt. Erst nach 18 Monaten konnte der Patient alle Aktivitäten wie vor dem Unfall wieder aufnehmen, außer Joggen über 15 min. Unbehagen auf dem Eis beim Schlittschuhlauf führten dazu, dass der Rennrodel-Sport nicht wieder aufgenommen wurde. Stattdessen wurden Kraft- und Ausdauersport sowie Fußballspielen möglich, was freizeitmäßig schmerzfrei ausgeübt wird. Gezielt zu Schmerzen befragt, werden Wetterfühligkeit und Berührungsempfindlichkeit der Narben am oberen Fußrücken angegeben. Seine handwerkliche Tätigkeit als Lackierer-Meister im 3‑Schicht-Betrieb kann er uneingeschränkt ausüben, auch verspüre er keine Einschränkung der Beweglichkeit des linken Fußes und seiner Zehen.

## 18-Jahres-Ergebnis

**Abb. 5:**
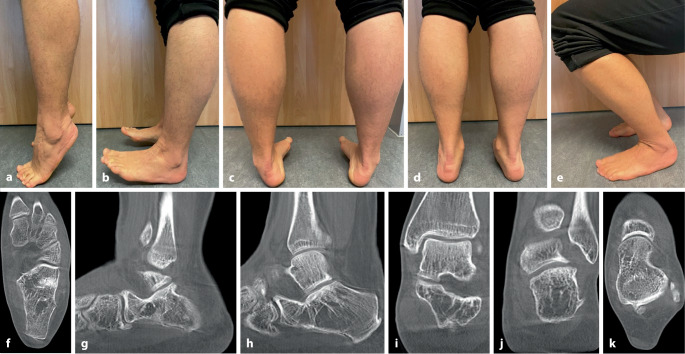
18-Jahres-Follow up: *Klinisch *sind Zehenspitzen- und Fersenstand (**a,b**), Inversion und Eversion (**c,d**) seitengleich aktiv ausführbar. Bei maximaler Kniebeuge (**e**) ist kein Einkrallen der Zehen links feststellbar. *Radiologisch *zeigt das CT im Stehen: Das linke Fersenbein hat eine normale, achsengerechte Form (**f**). Das Kalkaneokuboidalgelenk (**g,h**) hat normale Weite und ist nahezu völlig kongruent. Das Kuboid ist weiter medial (**h**) gering verkürzt fehlverheilt und plantar nach posterior ausgezogen. Die mediale Facette (**i**) ist normal weit, das Sustentaculum tali anatomisch verheilt. Die posteriore Facette (**j**) ist nahezu anatomisch verheilt, die subtalare Gelenkweite (**h,j**) normal. Der Talus (**k**) ist in Achse und Form normal verheilt, ebenso das nicht gezeigte Metaltarsale V

*Klinisch* zeigen sich keine Achsenabweichungen, ein plantigrader Fuß, volle Funktionen des OSG für Streckung und Beugung, des Fußes für Eversion und Inversion sowie für Pronation und Supination als auch voll flexible Zehen aktiv und passiv links (Abb. 5a–e). Die Narbe am Fußrücken nach Fasziotomie ist berührungsempfindlich, ein Hoffmann-Tinel-Zeichen ist nicht auslösbar. Ein schmerzfreies Hervortreten der Extensorensehnen bei aktiver Dorsalextension im OSG ist sichtbar (Abb. 5b). Die seitliche Fußnarbe ist kaum sichtbar und minimal berührungsempfindlich. Der American Orthopaedic Foot & Ankle Society (AOFAS) Ankle Hindfoot Score [11] beträgt 100, der Foot Function Index (FFI) 2,36 [3] und der EuroQol (EQ)-5L5D-Score [4] 100 Punkte.

*Radiologisch* zeigt das CT im Stehen unter Körperlast (Abb. 5f–k) nahezu Normalbefunde. Das linke Fersenbein hat eine achsengerechte und normale Form (Abb. 5f, h, j). Das Kalkaneokuboidalgelenk (Abb. 5g, h) hat eine normale Weite und ist nahezu völlig kongruent, allerdings ist das Kuboid weiter medial (Abb. 5h) gering verkürzt verheilt und plantar nach fersenwärts ausgezogen. Die mediale Facette des Kalkaneus (Abb. 5i) ist normal weit, das Sustentaculum tali anatomisch verheilt. Die posteriore Facette (Abb. 5j) ist nahezu anatomisch verheilt, die subtalare Gelenkweite (Abb. 5h, j) normal. Der Talus ist achsen- und formgerecht verheilt (Abb. 5k).

## Diskussion

### Epidemiologie eines akuten Fuß-Kompartmentsyndroms bei Kindern

Ein akutes Fuß-KS bei Kindern (2 bis 14 Jahre) wird mit einer extrem niedrigen Prävalenz von 0,00002 % [5] angegeben. In einer 2025 erschienenen systematischen Übersicht mit weltweiter Analyse bis 2024 betraf diese Diagnose in 5 von 38 Fallberichten ein Kind [26], in einer Serie von 8 Fällen mit kombiniertem Unterschenkel- und Fuß-KS nur ein einziges Kind [13]. Mortensen et al. [16] fanden in einer Metaanalyse für die gesamte untere Extremität eine Prävalenz von 0,3 %, Ahmed und Kuo [1] 1,1 % vorwiegend bei Tibiaschaftfrakturen und gehäuft bei additiver Gefäß-Nerven-Verletzung oder beim Crush-Trauma. Nach einer 20-Jahres-Analyse [2] eines Level-I-Trauma-Zentrums wurden 67 akute KS der unteren Extremität an 56 Kindern und Jugendlichen, davon 73,2 % männlich, versorgt. Davon waren Kinder bis 12 Jahre in 23,3 % betroffen, der Oberschenkel in 9,0 %, der Unterschenkel in 67,1 % und der Fuß nur in 23,9 %. Livingston et al. [12] beobachten unter 39 Fällen eines akuten KS im Kindesalter eine Diagnosefindung im Mittel nach 48 h (9–96 h), sodass bei Operation in 54 % bereits irreversible Muskelnekrosen bestanden und 31 % persistierende neurologische oder funktionelle Defizite hatten. Deshalb warnten sie vor dem Übersehen eines akuten KS bei bewusstlosen, intubierten Kindern oder regional betäubter Extremität und forderten die frühe (< 8h) Entlastung.

Nach der eingesehenen Literatur stellt die vorliegende Fallbeschreibung eines komplexen Fußtraumas mit seriellen Frakturen des Talus, Kalkaneus, Kuboids und des Metatarsale V sowie assoziiertem akuten Fuß-KS beim Kind die erste ihrer Art dar.

### Methodik der Fuß-Kompartment-Entlastung

In einer systematischen Übersicht von 5 Fallbeschreibungen eines akuten KS bei Kindern werden in Analogie zum Erwachsenen mediale, laterale und einfache sowie doppelte dorsale Inzisionen beschrieben [2]. Grundsätzliche Übereinstimmung besteht darin, dass eine Fasziotomie immer und zum möglichst frühen Zeitpunkt durchgeführt werden soll [5–8, 16, 28]. Zur Kompartmentspaltung bei Kalkaneusfraktur werden mediale, dorsale und kombinierte Zugänge beschrieben [5, 6, 13, 15, 22]. Im eigenen Vorgehen hat sich der „Hannover-approach“ auch beim Kind bewährt [20, 32]. Dieser wurde bei akutem Fuß-KS aufgrund komplexer Chopart‑/Lisfranc-Frakturen [24] initiiert, später auch bei Kalkaneusfrakturen angewandt [31]. Eine effektive Entlastung der Fußkompartimente bei Kalkaneusfraktur ist auch über einen medialen (modifizierten Henry‑)Zugang effektiv möglich [6, 13, 15, 22, 26]. Bei der dorsalen Fasziotomie sollte, ggf. durch intraoperative Druckmessung, sichergestellt werden, dass das plantar gelegene Kalkaneuskompartment sicher entlastet ist. Wenn nicht, muss eine zusätzliche mediale Fasziotomie erfolgen [15, 22]. Ein Vorteil beider Verfahren ist, dass die Verwendung eines späteren (ausgedehnt) lateralen Zuganges hierdurch nicht kompromittiert wird. Da die Faszien im Fuß schwächer ausgeprägt sind als am Unterschenkel, können diese insbesondere bei multiplen Frakturen, wie im vorgestellten Fall, bereits durch das Trauma zerrissen sein. Eine chirurgische Durchtrennung aller Faszien ist daher nicht immer erforderlich, eine suffiziente Entlastung aller Fußkompartimente muss jedoch, ggf. durch weitere Inzisionen sichergestellt sein [10].

Bei isolierter Kalkaneusfraktur des Erwachsenen mit akutem KS kann bei sofortiger Frakturversorgung in ausgewählten Fällen durch die Reduktion des Fragmentdruckes von innen und die Ausräumung des Hämatoms auch ein primärer Wundverschluss nach Frakturversorgung erfolgen [19, 31].

### Folgen eines Fuß-KS

Unterschiedlich sind die Angaben zu Folgen eines akuten Fuß-KS. So werden in 7 von 38 Fallbeschreibungen (18 %) bei Kindern Schmerzen, Parästhesien, Nervenläsionen oder Zehenkontrakturen angegeben [25, 26], wohingegen in 6 von 7 Kohortenstudien mit insgesamt 69 Fällen aller Altersgruppen Folgen wie Krallenzehen in 58 % [14] und plantare Parästhesien in 30 % [6] beschrieben werden. In einer prospektiven Studie sahen Han et al. [8] in 14 Fällen eines akuten KS des Fußes bei Erwachsenen, die zwischen 3 und 11 h nach Trauma entlastet wurden, dass nur 11 von 14 Patienten (78,6 %) wieder arbeitsfähig wurden, 28,6 % spezielle Schuhe oder Einlagen aufgrund sensorischer Defizite (in 21,4 %) oder bei Krallenzehenausbildung (in 14,3 %) benötigten. Diese Ergebnisse sahen die Autoren insbesondere in Fällen der späten Entlastung nach 6 h, weshalb sie die frühe, notfallmäßige Operation fordern. Eine Analogie hierzu findet sich bei Manoli et al. [13], die in 8 Fällen eines akuten kombinierten Unterschenkel- und Fuß-KS nur in 2 Fällen eine folgenlose Abheilung sahen. Das einzige Kind in dieser Serie, das 10-jährig als Fahrradfahrer vom Auto angefahren wurde, neben einem Abdominaltrauma eine Unterschenkelfraktur mit Unterschenkel-Fuß-KS erlitt und eine Kompartmentspaltung erst nach 24 h erhielt, entwickelte nach 3 Monaten Nekrosen des M. abductor hallucis, des M. flexor hallucis brevis und der interossären Fußmuskeln. Die eigene Erfahrung in 4 Fällen eines akuten Fuß-KS im Alter zwischen 9 und 11 Jahren [32] zeigt die Bedeutung des Zeitfaktors, da drei dieser Kinder innerhalb 6 h nach Trauma, eines nach 7 h entlastet wurde und alle bis auf eine Narbenempfindlichkeit keine weiteren Folgen des KS aufwiesen.

## Fazit für die Praxis

Bei Hochrasanztraumen mit akutem Kompartmentsyndroms des Fußes besteht beim Kind wie beim Erwachsenen eine notfallmäßige Indikation zur Dermatofasziotomie innerhalb von 6 h nach Trauma. Bei zusätzlicher Kalkaneusfraktur ist eine anatomische Rekonstruktion zu fordern, wobei wegen des Weichteilschadens in drei Schritten vorgegangen werden sollte. Die Osteosynthese sollte dem „Mini-Max-Prinzip nach B.G. Weber“ folgen, d. h. maximale Stabilität der reponierten Fraktur mit minimaler Implantatlast. Beim bewusstlosen Kind mit Fußfraktur(en) und massiver Weichteilschwellung ist ein KS durch wiederholte oder kontinuierliche Messungen auszuschließen. Wie beim Erwachsenen kann auch beim Kind der Druck für die vulnerable Fußbinnenmuskulatur, nicht erst ab 30 mm Hg, sondern bereits ab 25 mm Hg pathologisch sein.

## Data Availability

Alle dieser Arbeit zugrunde liegenden Daten sind in diesem Artikel enthalten.
